# Supplementary material for: Electrospinning and Rheological Characterization of Polyethylene Terephthalate and Polyvinyl Alcohol with Different Degrees of Hydrolysis Incorporating Molecularly Imprinted Polymers
Source: Polymers (Basel). 2024 Nov 26;16(23):3297. doi: 10.3390/polym16233297 (PMC11644715; doi:10.3390/polym16233297)
Supplement: Supplementary file 1 [file polymers-16-03297-s001.zip › polymers-3279061-supplementary.pdf]

Electrospinning and Rheological Characterization of Polyethylene Terephthalate and Polyvinyl Alcohol with Different Degrees of Hydrolysis Incorporating Molecularly Imprinted Polymers

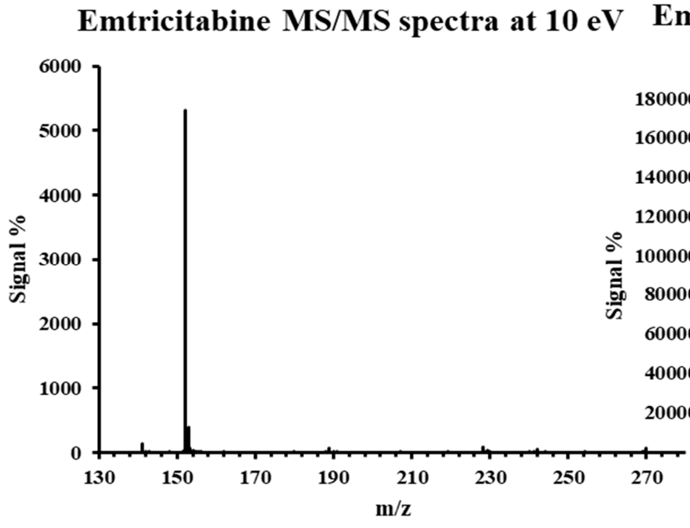
Emtricitabine MS/MS spectra at 10 eV
Signal %
m/z

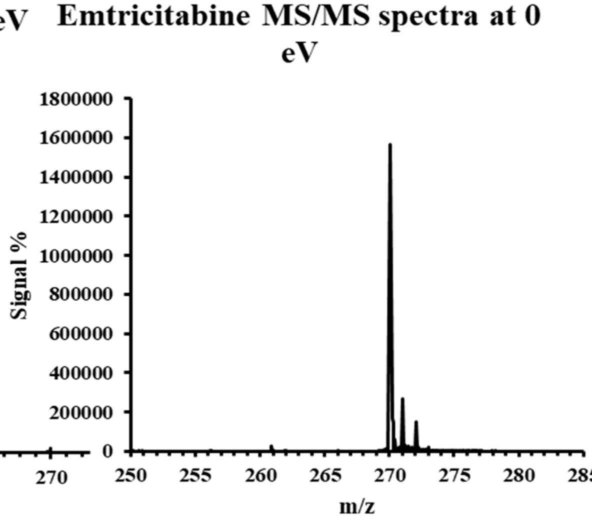
Emtricitabine MS/MS spectra at 0 eV
Signal %
m/z

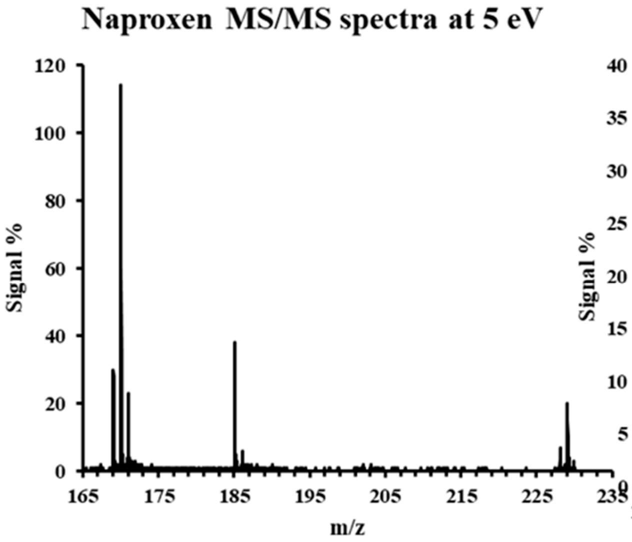
Naproxen MS/MS spectra at 5 eV
Signal %
m/z

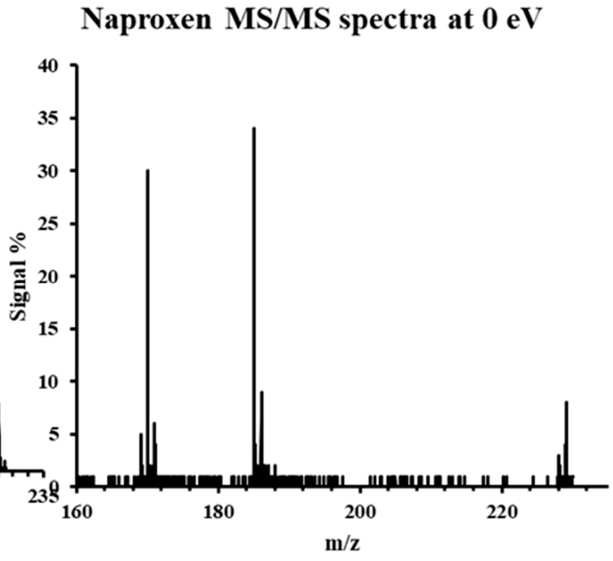
Naproxen MS/MS spectra at 0 eV
Signal %
m/z

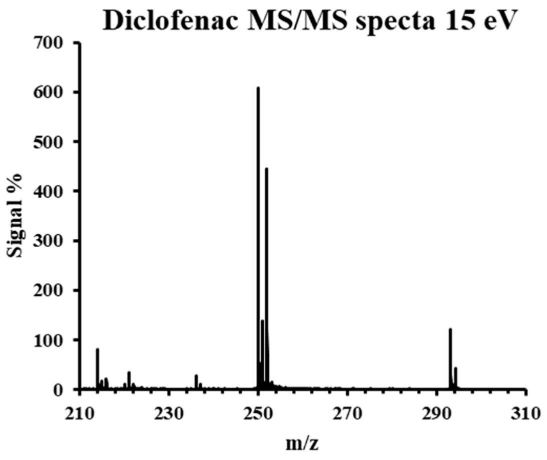
Diclofenac MS/MS specta 15 eV
Signal %
m/z

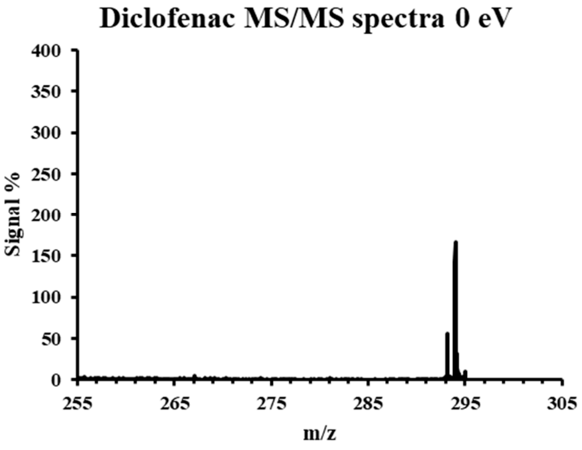
Diclofenac MS/MS spectra 0 eV
Signal %
m/z

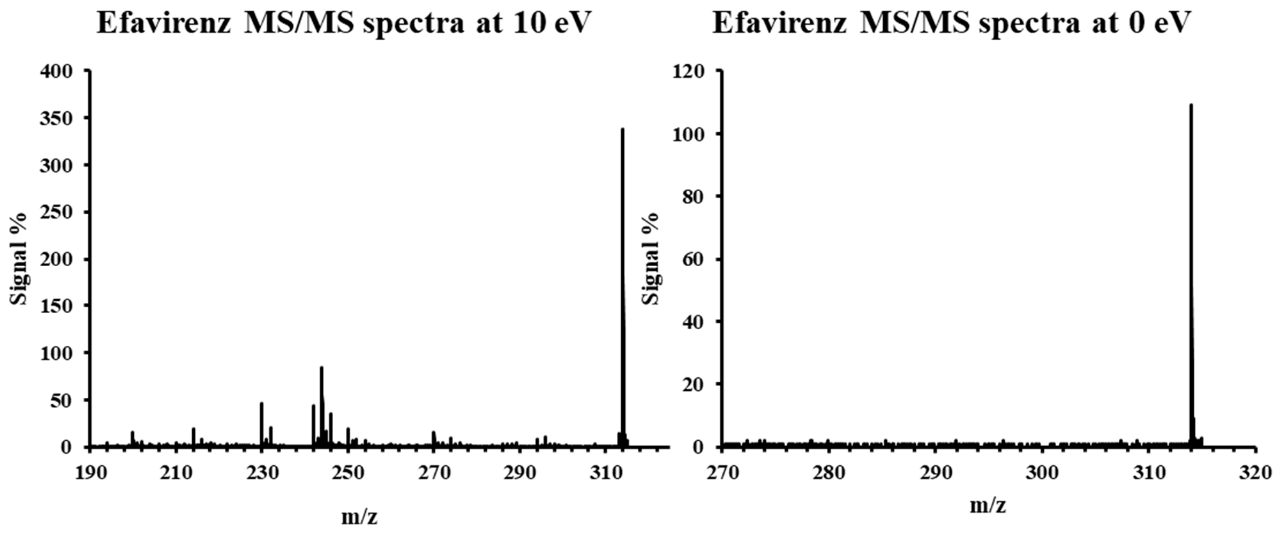

Figure S1. Comparison of MSMS optimization spectra of compounds at the chosen energy collision energy and of 0eV experiments courtesy of Emilie Drouin.

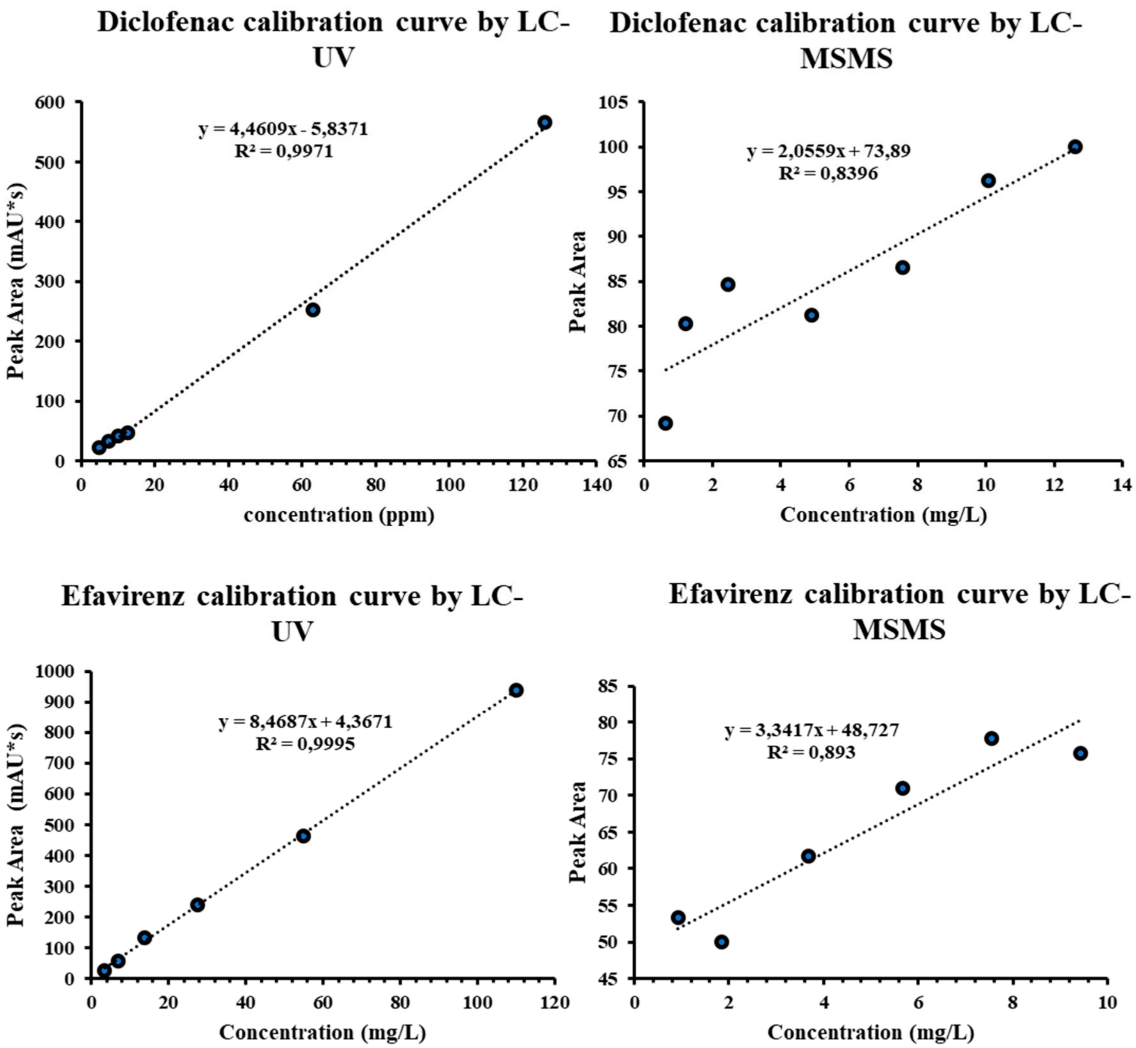
Diclofenac calibration curve by LC-UV
y = 4,4609x - 5,8371
R² = 0,9971
Peak Area (mAU*s)
concentration (ppm)
Diclofenac calibration curve by LC-MSMS
y = 2,0559x + 73,89
R² = 0,8396
Peak Area
Concentration (mg/L)
Efavirenz calibration curve by LC-UV
y = 8,4687x + 4,3671
R² = 0,9995
Peak Area (mAU*s)
Concentration (mg/L)
Efavirenz calibration curve by LC-MSMS
y = 3,3417x + 48,727
R² = 0,893
Peak Area
Concentration (mg/L)

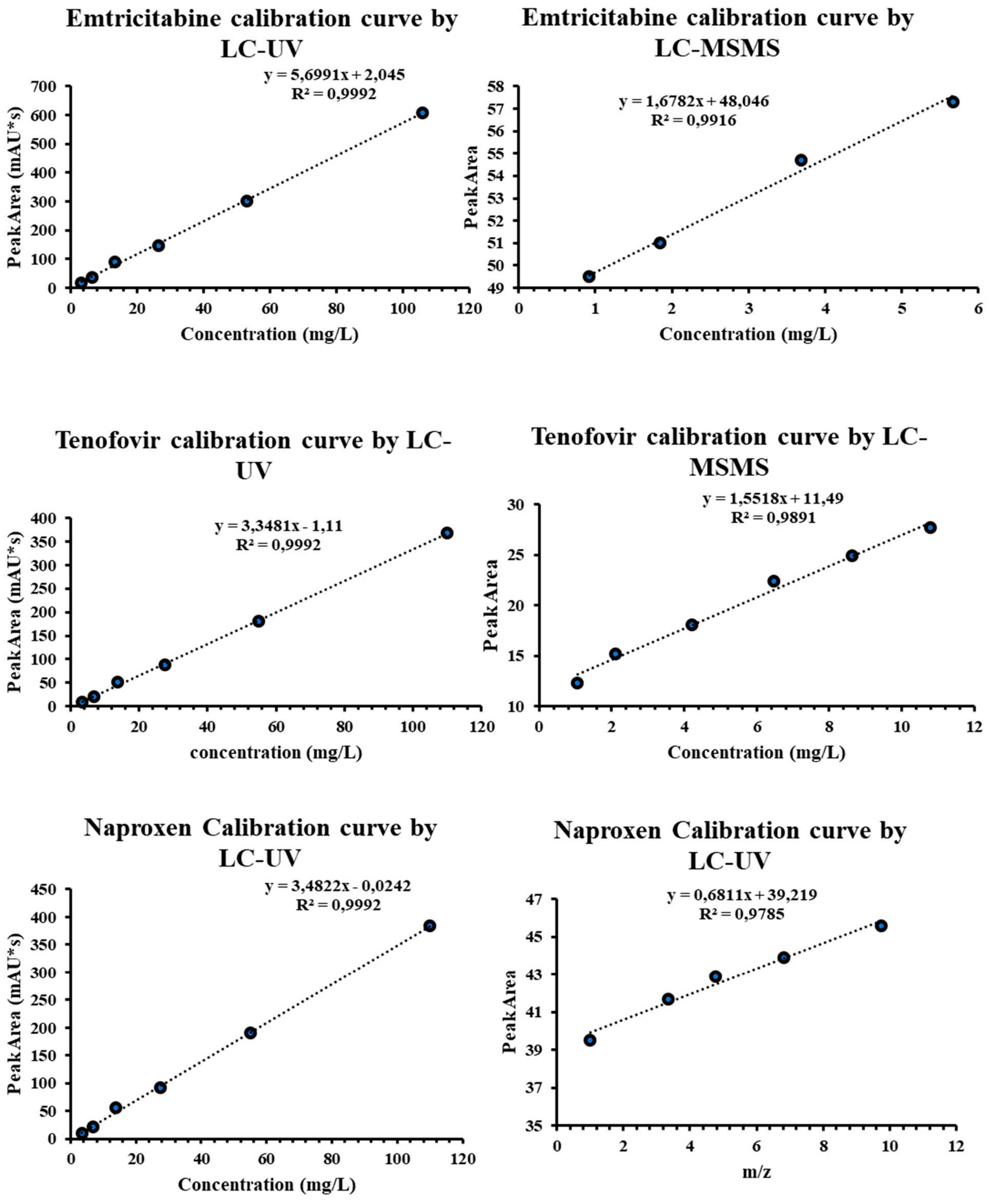

Figure S2. Comparative calibration curves of target compounds by LC-UV and LCMSMS experiments courtesy of Emilie Drouin.

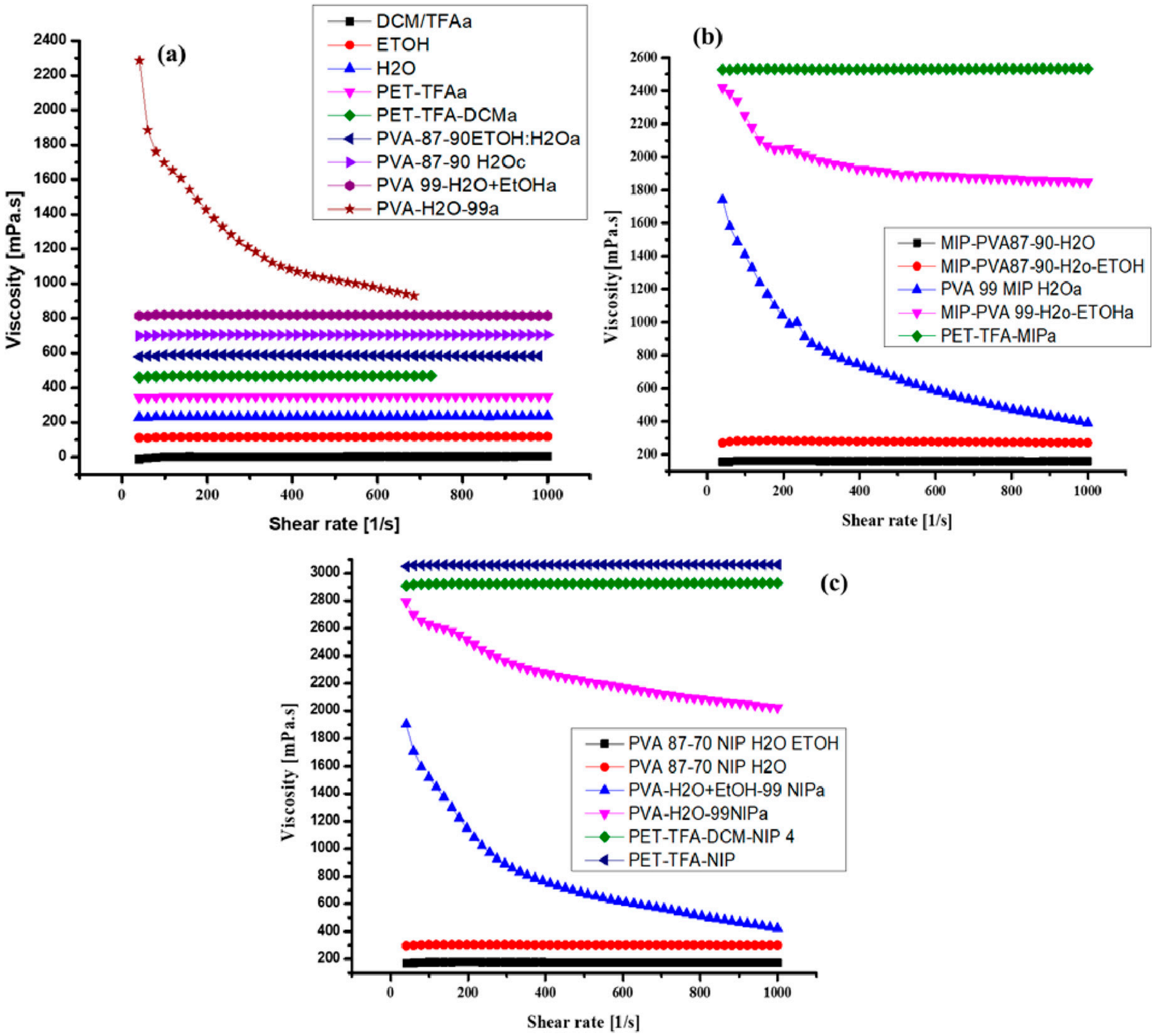

Figure S3. Shear vs viscosity of Solvents, Pure Polymers solutions, Polymer solutions with MIP and NIP.

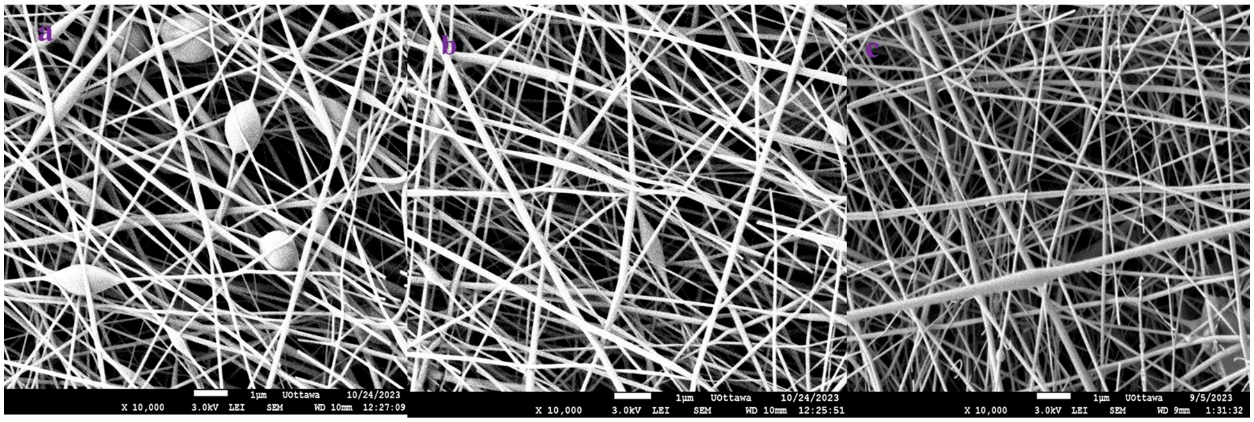

Figure S4. PVA H2O SEM images, Flowrate = 0.3 mL/min^{-1}, TCD= 15 cm, needle= 11 G, Voltage (a) 10kV, (b) 15 kV, (c) 18 kV.

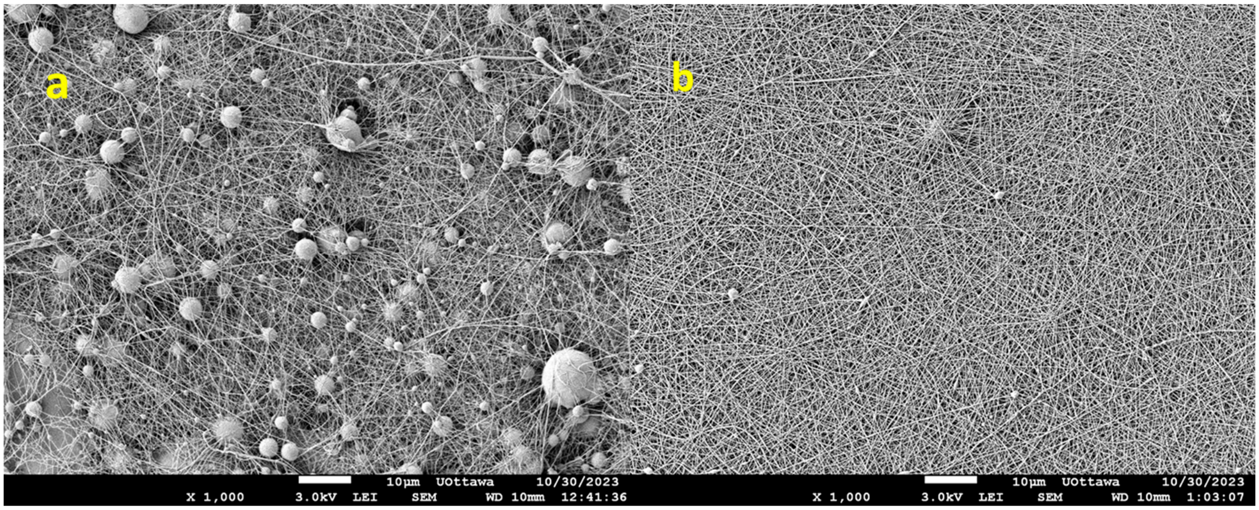

Figure S5. PET/TFA(a) and PET/TFA/DCM (b).
